# Rare Cause of Syncope in a Gravid Female

**DOI:** 10.5811/cpcem.2020.5.46948

**Published:** 2020-07-14

**Authors:** Andrew Bellino, Katherine Staats, Jessica Ngo

**Affiliations:** * Stanford University School of Medicine, Emergency Medicine Residency Program, Stanford, California; † Stanford University School of Medicine, Department of Emergency Medicine, Stanford, California

**Keywords:** splenic artery aneurysm, aneurysm rupture, pregnancy, syncope

## Abstract

**Case Presentation:**

A 33-year-old gravid female was brought to the emergency department after she collapsed in the street. Point-of-care ultrasound showed free fluid in the abdomen and confirmed an intrauterine pregnancy. Surgical teams were consulted, and cross-sectional imaging revealed a spontaneously ruptured splenic artery aneurysm (SAA). The patient was taken expeditiously to the operating room for splenic artery ligation and subsequent splenectomy.

**Discussion:**

Ruptured SAA in pregnant patients is associated with significant mortality for both mother and fetus. Maintaining a high index of suspicion in the correct population is crucial to avoid diagnostic errors and provide definitive care with operative repair.

## CASE PRESENTATION

**Image 1:**
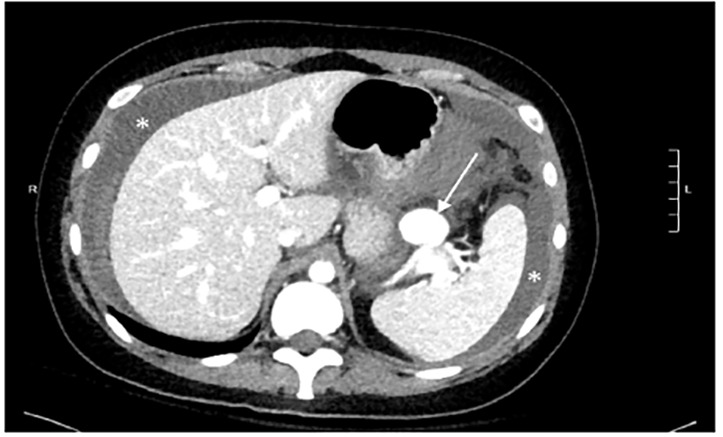
Computed tomography axial section showing a large splenic artery aneurysm (arrow). There is also significant hemoperitoneum surrounding the liver and spleen (asterisks).

**Image 2:**
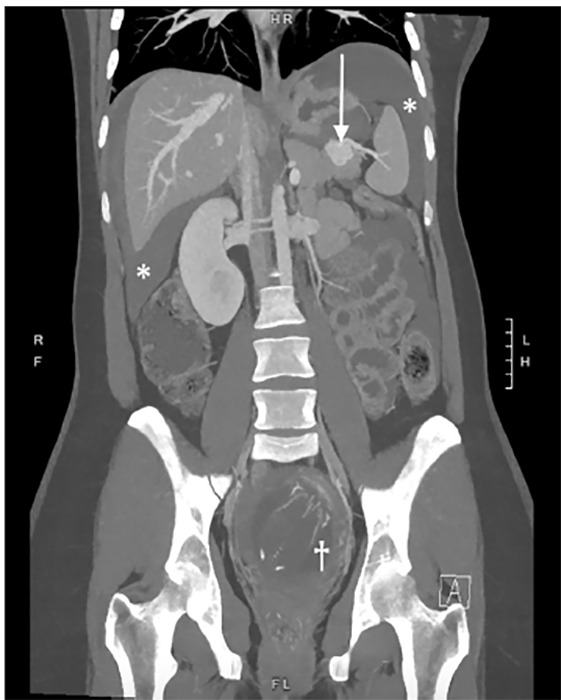
Computed tomography coronal reformat showing the ruptured splenic artery aneurysm (arrow) and significant hemoperitoneum around the liver and spleen (asterisks). The gravid uterus with developing fetus is also noted within the pelvis (dagger), a combination rarely seen on imaging due to the high mortality of this disease.

A 33-year-old gravida 1 para 0 at 18 weeks gestational age presented to the emergency department for syncope. The patient had passed out while crossing the street and emergency medical services were activated. She reported severe abdominal pain after arrival and vitals showed a heart rate of 120 beats per minute and a blood pressure of 88/52 millimeters of mercury. Point-of-care ultrasound showed free fluid in the left upper quadrant and confirmed an intrauterine pregnancy with good cardiac activity. Obstetrics and general surgery teams were consulted. Following improvement of the patient’s vital signs with a crystalloid bolus, a computed tomography was performed, which revealed a spontaneously ruptured and previously undiagnosed 2.6-centimeter splenic artery aneurysm (SAA) (Images 1 and 2).

The patient was taken emergently to the operating room where surgeons evacuated six liters of blood that originated from her splenic artery rupture. Splenectomy was successful in stabilization; however, post-operatively no fetal heart rate was found and a dilation and evacuation was subsequently performed. The patient was discharged home on day 14.

## DISCUSSION

The true incidence of SAA is unknown; however, estimates range from 0.02–10.4%.^1^,^2^ Of those diagnosed, ruptured aneurysm is only seen in 5% of cases, and it is associated with high mortality.^2^,^3^ SAA is more common in females (4:1) and is associated with pregnancy, hypertension, connective tissue disease, portal hypertension, and atherosclerosis.^1^ Prophylactic treatment of unruptured aneurysm is recommended for women of childbearing age due to increased risk of rupture in pregnancy.^2^,^4^ Unfortunately, given its low incidence, ruptured aneurysm is often mistaken for more common pregnancy-related pathologies such as ruptured ectopic pregnancy, placental abruption, uterine rupture, pulmonary embolism, and perforated peptic ulcer.^4^ Prompt diagnosis and treatment with endovascular or open technique is crucial for maternal and fetal survival as mortality rates approach 70% and 90%, respectively.^5^

**CPC-EM Capsule**

What do we already know about this clinical entity?

Splenic artery aneurysms are usually asymptomatic until ruptured at which point they are associated with high mortality. Pregnant women are at increased risk.

What is the major impact of the image(s)?

Images show ruptured aneurysm in conjunction with the developing fetus. These images are uncommon as such patients are often too unstable for advanced imaging.

How might this improve emergency medicine practice?

Understanding this deadly disease can improve emergency physicians’ ability to quickly make the diagnosis and initiate effective treatment.
